# [Retracted] β-asarone relieves chronic unpredictable mild stress induced depression by regulating the extracellular signal-regulated kinase signaling pathway

**DOI:** 10.3892/etm.2026.13063

**Published:** 2026-01-12

**Authors:** Haiying Dong, Weiliang Cong, Xiwen Guo, Yuhua Wang, Shengju Tong, Qiang Li, Chengchong Li

Exp Ther Med 18:3767–3774, 2019; DOI: 10.3892/etm.2019.8018

Following the publication of the above paper, it was drawn to the Editor’s attention by a concerned reader that certain of the flow cytometric data shown in Fig. 3 on p. 3770 were strikingly similar to data that had already been submitted to other journals in papers written by different authors at different research institutes.

In view of the fact that the abovementioned data had already apparently been submitted for publication prior to its submission to *Experimental and Therapeutic Medicine*, the Editor has decided that this paper should be retracted from the Journal. The authors were asked for an explanation to account for these concerns, but the Editorial Office did not receive a reply. The Editor apologizes to the readership for any inconvenience caused.

